# Supplementary material for: The “Alternative” Choice of Constitutive Exons throughout Evolution
Source: PLoS Genet. 2007 Nov 16;3(11):e203. doi: 10.1371/journal.pgen.0030203 (PMC2077895; doi:10.1371/journal.pgen.0030203)
Supplement: Supplementary file 1 [file pgen.0030203.sd001.doc]

**Comparative analysis of 5'ss**

The major changes in the 5'ss between conserved constitutively and alternatively spliced exons are the decrease in the total percent of 'A' and 'G' in position 3 of the 5'ss: 94.8% of the nucleotides in that position are either 'A' or 'G' (61.9% are A and 32.9% are 'G') in conserved constitutively spliced exons, whereas 91.1% of the nucleotides of conserved alternative exons are 'A' or 'G' (66.6% are A and 24.5% are G)(χ^2^, P value (A+G) = 7.14E^-05^). Examination of this position in species specific exons revealed that they exhibit 'A' or 'G' in 94.28% of the cases, which is similar to constitutive exons and differs significantly from alternatively spliced ones (χ^2^, P value (A+G) = 0.033). In position 4 of the 5'ss the dominant 'A' with 70.1% frequency in the conserved constitutive exons is decreased to 62.4% in the conserved alternative exons (χ^2^, P value = 4.80E^-05^), this is linked to an increase in 'G' at that position (from 10.9% to 14.1% in constitutive and alternative exons; χ^2^, P value = 1.36E^-02^) and in 'T' (from 11.8% in the constitutive to 15.3% in the alternatives; χ^2^, P value = 8.87E^-03^). Therefore, the change in position 4 from 'A' to 'G' or 'T' between constitutive to alternative spliced exons replaces a canonical base pairing with non-canonical base pairing, and thus weakening the U1/5'ss binding. Inspecting this position in species specific exons revealed the frequencies of the nucleotides 'A', 'G' and 'T' to be 67.65%, 10.78% and 13.73%, respectively, which does not differ significantly from either type of exons. The last position that shows a statistically significant change between conserved constitutively and alternatively spliced exon is position 5. We found a decrease in 'G' from 78.1% in conserved constitutive exons to 71.5% in conserved alternative exons (χ^2^, P value = 1.17E^-04^), and an increase in 'A' in position 5 from 8.9% in conserved constitutive exons to 12.4% in conserved alternative ones (χ^2^, P value = 2.68E^-03^). When we tested position 5 in species specific exons we found the frequency of nucleotides 'G' and A to be 82.03% and 7.68%, respectively. These results differ significantly from alternative exons (χ^2^, P value (A) = 1.40E^-05^ ; P value (G) = 6.12E^-03^) but not from constitutively spliced ones (Fig. S1).

In 12 out of 15 tested parameters in Fig. 1A the species-specific spliced exons (ssEx) could not be subdivided to ssEX-alternative and ssEX-constitutive. However, in the average introns length we revealed a significant difference, where the ssEX-alternatively spliced exons are flanked by longer introns compared to ssEx-constitutive. In the ΔG we did not find statistically significant differences between ssEx-alternative and ssEx-constitutive (data not shown).
