# Supplementary material for: The “Alternative” Choice of Constitutive Exons throughout Evolution
Source: PLoS Genet. 2007 Nov 16;3(11):e203. doi: 10.1371/journal.pgen.0030203 (PMC2077895; doi:10.1371/journal.pgen.0030203)
Supplement: Supplementary file 2 [file pgen.0030203.sd002.doc]

**Note:** The difference in the number of orthologous exons analyzed between Fig. 4a and 4b stems from the fact that the downstream intron is missing in the non-vertebrate species, due to intron gain/loss.

**The differences in wt between mouse and other species in Fig. 4c**

The inclusion level of the wt exon is similar in human, chicken, and zebrafish (~20%). It is surprising that this inclusion level is significantly different from that of mouse (63%; Fig. 4c, lane 1 in all panels). High inclusion level was also observed when a different mouse cell line was used (DA-3: mammary carcinoma cell-line, not shown). The ESRs and the 5'ss are the same in this experiment. Therefore, the difference must reside in the different cellular environment between the mouse cell lines and the other organisms. This either may be the result of evolutionary changes in the lineage leading to mouse that caused different function of the mouse splicing factors, or may reflect different levels/modifications of splicing regulatory proteins.
