# Supplementary material for: The “Alternative” Choice of Constitutive Exons throughout Evolution
Source: PLoS Genet. 2007 Nov 16;3(11):e203. doi: 10.1371/journal.pgen.0030203 (PMC2077895; doi:10.1371/journal.pgen.0030203)
Supplement: Supplementary file 3 [file pgen.0030203.sd003.doc]

**The functionality of the exon skipping isoforms of the species-specific spliced exons.** Out of 354 human species specific exons that are alternatively spliced in human and constitutively spliced in mouse: (1) 11 exons are found within the UTR (2) 343 exons are in the CDS, of which 207 exons are within the CDS are nonsymmetrical, and 136 exons are symmetrical (3) For 102 human exons a protein containing the exon (exon inclusion) was found in Uniprot database (Swissprot+Trembl), and for 17 proteins the exon skipping isoform was also found (all 17 cases are of symmetrical exons). Out of 258 Mouse species specific exons that are constitutively spliced in human but alternatively spliced in mouse: (1) 5 are found within the UTR (2) All others are in the CDS, of which w 149 are non-symmetrical and 104 are symmetrical (3) For 8 exons proteins were found indicating the exon inclusion isoform, and for 2 exons the exon skipping isoforms were also found in Uniprot. Symmetrical exons position within the CDS shows equale distribution along the CDS, whereas showing a preference for non-symmetrical exons to located at the 5' half of the CDS (Mann-Whitney between symmetrical and non-symmetrical P value <0.01; same analysis as in Magen and Ast, 2005; see Fig. S2). This suggests that some of the new isoforms are already functional and contain a protein that is synthesized from the skipping isoform. Others might be functional in the future following mutations that will presumably generate a symmetrical exon without premature stop codon, and thus can causes further speciation. Whereas the rest might be returned back to the constitutive splicing state.

**Fig. S2**

Non-symetrical exons position in the CDS:
