# Supplementary material for: The “Alternative” Choice of Constitutive Exons throughout Evolution
Source: PLoS Genet. 2007 Nov 16;3(11):e203. doi: 10.1371/journal.pgen.0030203 (PMC2077895; doi:10.1371/journal.pgen.0030203)
Supplement: Supplementary file 4 [file pgen.0030203.sd004.doc]

**Minigenes' sequences:**

Exons and introns sequences are in uppercase and lowercase, respectively. Sequences in red indicate the alternative exon. In IMP minigene, the red exon is constitutively spliced but branch-site mutation (A->G colored green) turned it to alternatively spliced exon.

**SLC35B3**

GAATTAATATTTTCAGTGGAGGGTTTTAAGTCCTGTGGCTGGTACCTTACCTTAGTGCAGTTTGCCTTTTACTCCATATTTGGCCTAATAGAACTTCAGCTTATTCAGGACAAAAGGAGGAGgtatgtggtttgtttttattttttgatatttctatattagttgaatttttttggcatactgaatatgcatgaaatttatacttctattaacaaaacatgtgataggcacgaactcttagtgtaactaacttaatactaaatacattttattcatttatacactgaaaaaaataaagtcttaaatgcagtattacaaaataaaaaaatattagctttaggctcaacttagtgttatatgttcttacaacatgataaaaaacacaatgtacatacttgatttgtataaattctgctgaaataggtgcagttactgatttgtagtacaactaacatcacagtggtgaaatgaaatatcagtacaggttgagcatcctaatccaaaaatcctaaatcc+ecoricttgggttattttagcagaatggatagatgtatttttcctaatgctagatggaaacattaggaaacagaacaacaattcaagagttaaaaattagttttaaaatgttaaggtaccttttaccattatggaggccaacattagctgggcatggtggcgcacacctgtagtcccagctactcaggaggctgaggcaaaagaatttcttgaacccgtgaggcagaggttgcacttagctgagatcatgccactgcactccagcctaggcaacagagcgagaccttatctcaagcaaacaacaacaaaagaagctctttttctctaacttaagatggttaagatggtttttatttgtggctagaactgctgttaaaaaaaaaaaaaaaaccttttgtcaacatttataccttcagtaaaaatctaaactcttttttacataattgagtggcctcgtttatgattgactaaataaaggtaaattgttatttttataaacttttttatacaagagcattacagtttttaaatagtgatattttttactgatatttccagaaatagtaaccagaaattagaaaagtaattagacatttacacaatgtgaacacattatcttttagtagtatgaattgggtcccaaaatgaaggttttaaaaattgtcttttacagAATACCAGGAAAAACCTACATGATAATAGCTTTTCTAACTGTGGGTACTATGGGGTTATCAAACACTTCCTTGGGCTACCTGAATTACCCTACCCAAGTCATCTTCAAGTGCTGCAAATTGATTCCTGTTATGCTAGGAGGAGTTTTTATTCAAGgtatagtaatgatatgtttttatactatttaaaactaataggctatgttatggcatctagcattaggaaaaacattaaacttctattacattttcaatgagcattctagatattgtaacaattctgaattatgtcttaccaaaataaggtattctaaaaacactgatggccaggcacagtggctcacgcctgtaatcccagcactgtgggatgccaaggtgggcagatcacaaggtcaggagttcgagaccagcctggccaatatggtgaaaccctgtctctactaaaaatacaaaaattagctaggcatggtggtgtatccatgtagtctcagctacttgggaggctgaggcagacgaattgcttgaacccaggaggcagaggttgcagtgagccgaggtcacaccactgcactccagcctgggcgacagagcgagactccatctcaaaacaaacaaacaaacaaacaaacaaacacttatatgccttagtttctgaagtcagtaatttggacttaagaaaaagaaaagataatactttgtttttattattttattttggaatctaccacatttccagcaatactatatttacgtattaaaggtctagttcaaaagtacgtggaaaggtcccttttttcagttccttcattcttctactctccaggggtctacccttcacttccattgtgcatgggcagttctcatattgaagtaaagtttaatctgagcagaactgcgtttttaccaacctgctggctttagtgatgtttagagccccagctaagtgacaagttttgtggaaatacgagtaaaagaggaggcagtgatagcaacaaacagtggaagtagcttctgttgttcacattaattgtcacgaggaatattcgtgttttaatagtcgggtgtttctgtatcactgaagttcttatacagcctgatatcatttatgcattagataccatcccagaataagcctttaaaaatgaaagtgttgtcaactacttcacgtatcatttcaggataacttcatataggaaaaaataattgacctcaatccctattaaaaatcacatttattgtcccaactgactaggcatatataatattgtcgttataaaccttacaaaaaagtcatactacattttatttttaaaataatgatatatgaaaataatttaattttcacctgcctggaagttatcacattatgatatttgcaaacttaaacagttatgtaaccagactcattaatctggttacttcgtaaatttacaaactacgagaaaattcataaccttttattatgcacaaagtaaaacaggaactaaatttccaggaaattttttaaatgtttgtggatgtgcattattcttgctgtcagtttttgccaaagtatgtattttagtgtcactattataccagataaagtagctctcgtttctaacttacagggacatattgctgcccattggtagttttcagaagccctcttgtgactgtcttctgccttgacctaagtgttcacgtttttggcattggatataaaaaaacaaatccatgttctacttaacacatagagcaaatataaagctgggtgggtatttgcataatgaagtggacttacgtttgttttatacagGAAAGCGTTATAATGTTGCAGATGTGTCTGCTGCCATATGTATGAGCCTTGGCCTGATATGGTTTACCCTCGCTGACAGCACAACTGCACCAAATTTCAACCTGACGG

**IMP**

CTGCAGTCTCACCTGAtCCCTGGCCTGAACCTGGCTGCTGTAGGTCTTTTCCCAGCTTCATCCAGCGCAGTCCCGCCGCCTCCCAGCAGCGTTACTGGGGCTGCTCCCTATAGCTCCTTTATGgtaggtggaagatcttattacacgggatccctgggcccatatgtggcctcccttaagggggactcagcatgctctgggagttggatgcttggcaagtttgcagggagagggaaggaaggtctcagaggcatccctcctctccagtgctcctagccactggctctgaggctcccaaatgtctgcctagaatgctcccggggcatagacagcactgttcccttactctctcctggcctcttccctttagccctactgggcataggaagagctaaagagctcacagcatcccttggcagcccagggtggcggtttggtgcctgtactccttcctccttgaggatgcaaggcaggggagtggagggtttctgggccatgctacttgggccccctggtaacaacgcctcctatcctggcagCAGGCTCCCGAGCAGGAGATGGTGCAGGTGTTTATCCCCGCCCAGGCAGTGGGCGCCATCATCGGCAAGAAGGGGCAGCACATCAAACAGCTCTCCCGGTTTGCCAGCGCCTCCATCAAGgtgatctgctctgtgggtcttcctgtttctggaaagggaggggtctctgcttttatcactctgcactttctcccatattccccctactcatttgagaattctgtgtgtcatatgagggacctttcccccatggaatcgaagtcctctttgtttttgatcttgcttggtccccatgggatggcttcagtgcttctgggttttcaggctgggcaggggcagggttataacatagaaagagaacttaggttctaacacaaactgatgggttcaaatccaagatccatctcatcaccccttcccccaatacactatatgatctgagcctcactttcctcgtcagtacaatattatgaggctacgcagccaaagcccttcgctcacatactaacaccaagtaagtgttagttgtctgtcaccttccctggccaaggtccaggggagagagaagggagaatctgtcatagtgaaacaaggatgctgttagagtttcacaaacctaaatccggttgctttagttgtcttaaaagctttgagcaaaaaccttgattgttctccagtggaggtgcagtcactgccctctatccgttggtcatttcatttgtgtccttgcctgttggcaagactccactgaaacctctctgggagattggtaggtggagggggcaggaggccctacttagaaagtgtcattgaagccaatccttctaactgaccacctctgccctcctaataattctggtgtgaaggcgtaatgatgtgggcttcagggtctttgttcttcctcccctaagtcttcagaatgggtagttgggagtaagggtggtagaaggggaactggatgaagtggacatggtgggggtcttcccatagagggtccctcattgactagctttttgctttttagATTGCACCACCCGAAACACCTGACTCCAAAGTTCGTATGGTTATCATCACTGGACCGCCAGAGGCCCAATTCAAGgttttggtctttattgttttccaagctgaacatggaggaattgaggttgggtatttcccctgaggtaggaaaaaggctgggtcagtttcccgttagctgtcaagtcctcatcacatctttaagccttccatgcaggataaagggctgcagagctattttcaaattgacatcaaactggatttctgttgacttcgtcttccctttttaaggtccacagaagaagatgggaaggaaagaagtctgagggcatcttatttgcactctgctgtcatttctaaggaaggcctttaatgccaaattctcatcttttatgtccccactaaatcctaaggttcttgaacttctgatcagacagccaaaaaatgaaccatcaactagcttaacctaacatatgtgaggatagaggactgggacagctctctgggccactggagagtcagacaggcctgccctctgtgtgacttgattagttgccattcagggccttgaaatgctggtcagtgccacgtccagattccataggtctggtttgtagcttgtgaattagattcctctgggaactattttttcccttgggaagtgtttattcaattactcataactcataatctccacccagggtctccagtatgatagctaagcagaaggaatgctgacaagctgcccacctgagccttgcccttcacctgccttagatggttagcccgcctaggatggggagggcctgtgggccagaaaatatgggctggagcttcctttttcccacttttctcttttgtcacagatatatgaaccactgattaacaattacctcctcttctcagGCTCAGGGAAGAATCTATGGCAAACTCAAGGAGGAGAACTTCTTTGGTCCCAAGGAGGAAGTGAAGCTGGAGACCCACATACGTGTGCCAGCATCAGCAGCTGGCCGGGTCATTGGCAAAGGTGGAAAAACG
