# Supplementary material for: The “Alternative” Choice of Constitutive Exons throughout Evolution
Source: PLoS Genet. 2007 Nov 16;3(11):e203. doi: 10.1371/journal.pgen.0030203 (PMC2077895; doi:10.1371/journal.pgen.0030203)
Supplement: Supplementary file 5 [file pgen.0030203.sd005.doc]

**The non-conserved spliced isoforms from Fig. 2:**

**DDO**: Rat upper PCR product could not be sequenced and no known ESTs or cDNAs supported this isoform; it is presumably a non-conserved, alternatively spliced exon.

**DPM1**: Human upper PCR product contains an exonized Alu. Accession no. CR598461 indicates that this isoform is biologically relevant.

**APGL5**: Rat band contains four exons, see NM_001014250 for this isoform. We used a primer complementary to the upstream exon for the PCR analysis because the genomic sequence of rat around the upstream exon was not fully mapped. Sequencing of the PCR product indicated that the upstream exon was present in the mRNA of rat.

**MTHFD2**: Human upper PCR product is present in accession no. NM_001040409 and is a non-conserved alternatively spliced exon. Rat upper PCR product is present in EST no. BP488687 and is a non-conserved alternatively spliced exon. Zebrafish PCR product contains four exons, see NM_001002181, and the splice pattern observed does not indicate non-conserved splicing; in this case we used a primer complementary to the next upstream exon for the PCR analysis, because the genomic sequence of the upstream exon was not fully mapped.

**SLC35B3**: Clean sequence was not obtained from upper PCR products from human, mouse, and rat; it seems as if the first exon was duplicated. Use of forward primers containing 15 nucleotides complementary to the end of the first exon and 15 nucleotides to the beginning of that exon confirmed that the upper PCR product contained exon duplication at the mRNA level, but not in the genomic sequence.

**SLC25A**: Human upper PCR product mRNA contains six exons, there is no single ESR/cDNA indicating inclusion of all these exons; however, different submissions show that they are real exons (CR749715, BC003399). The human lower PCR product contains the flanking exons and the tested alternative exon, this isoform is supported by ESTs BI825086 and BU663340. Chicken lower PCR product contains three exons, including the alternative tested; this isoform is supported by EST no. CK986868. The Xenopus PCR product represents amplification of three exons including the tested exon, support by mRNA no. BC074600.

**RABL5**: Rat lower band result of use of alternative 3'ss of the species-specific exon; supported by EST no. CB787434 (there are 10 similar ESTs). Chicken upper product results from intron retention (104 bp) between the first exon and the alternative tested exon; no submission that supports this isoform.

**DGCR6L**: Dog upper PCR product is supported by EST no. DN392941 representing a non-conserved alternatively spliced exon. The ortholog gene in zebrafish is not supported by a continuous stretch in the genome; this gene has sequence in two distinct locations, with the first ~400 nt present in chromosome 8 and the rest in chromosome 14. Primers used were complementary to a sequence in chromosome 8 and in chromosome 14. The presence of an RT-PCR product raises the possibility that this single mRNA is constructed from transcripts from two different chromosomes (trans-splicing). However, since the zebrafish genome is still provisional, there is a possibility for that gene duplication could explain this band as coming from a single chromosome.
