# Supplementary material for: The “Alternative” Choice of Constitutive Exons throughout Evolution
Source: PLoS Genet. 2007 Nov 16;3(11):e203. doi: 10.1371/journal.pgen.0030203 (PMC2077895; doi:10.1371/journal.pgen.0030203)
Supplement: Supplementary file 6 [file pgen.0030203.sd006.doc]

**Additional Materials and Methods**

**Splice site analysis**

The prediction of free energy was calculated using the OligoArrayAux 1.9 (Rouillard et al. 2002) that was developed by Nick Markham and Michael Zuker at the Rensselaer Polytechnic Institute. Among other things, the OligoArrayAux 1.9 predicts the free energy (ΔG) of a single folded RNA strand. To predict the ΔG of two short RNA strands, we concatenated these strands into one RNA strand as follows: “NN”-Seq1-“NNNNN”-reversed U1 (UACUUACCUG)-“NN”. The software does not hybridize the “N” sequence (because it is considered neutral). Therefore, the resulting calculation of free energy is that of the U1:5'ss hybrid. The pictograms were done using the pictogram server (http://genes.mit.edu/pictogram.html). We detected and analyzed the polypyrimidine tract (PPT) and branch site sequence (BSS) using the algorithm previously described by (Kol et al. 2005). In this analysis, the restrictions were modified to allow detection of PPT containing less than 14 base pairs.

**Fish maintenance**

Adult Zebrafish were raised in a recirculation water system under optimal conditions: 12 hours light and 12 hours dark cycles, at 28°c and fed twice a day (Whitlock and Westerfield 2000). To produce embryos, male and female zebrafish were paired in the evening, and spawning occurred within 1 hour after the lights were turned on. Embryos were immediately used for microinjection.
