# Supplementary material for: The “Alternative” Choice of Constitutive Exons throughout Evolution
Source: PLoS Genet. 2007 Nov 16;3(11):e203. doi: 10.1371/journal.pgen.0030203 (PMC2077895; doi:10.1371/journal.pgen.0030203)
Supplement: Supplementary file 7 [file pgen.0030203.sg001.doc]

**Fig. S1. The major changes in the 5'ss between conserved constitutive, alternative and species-specific exons.**

The consensus 3' (left-upstream intron) and 5' (right-downstream intron) splice sites sequences of constitutive, alternative and species-specific exons were extracted and was then represented graphically using the pictogram server (Burge CB <http://genes.mit.edu/pictogram.html>).
