# Supplementary material for: The “Alternative” Choice of Constitutive Exons throughout Evolution
Source: PLoS Genet. 2007 Nov 16;3(11):e203. doi: 10.1371/journal.pgen.0030203 (PMC2077895; doi:10.1371/journal.pgen.0030203)
Supplement: Supplementary file 8 [file pgen.0030203.sg002.doc]

**Fig. S2 - Symmetrical exons position within the CDS shows equale distribution along the CDS, whereas showing a preference for non-symmetrical exons to located at the 5' half of the CDS**

Non-symetrical exons position in the CDS:
