# Supplementary material for: The “Alternative” Choice of Constitutive Exons throughout Evolution
Source: PLoS Genet. 2007 Nov 16;3(11):e203. doi: 10.1371/journal.pgen.0030203 (PMC2077895; doi:10.1371/journal.pgen.0030203)
Supplement: Supplementary file 9 [file pgen.0030203.sg003.doc]

**Fig. S3 –zebrafish-GFP**

**Y**

**H**

**T**

**CMV promoter-driven transient expression of wt SLC35B3 minigene in zebrafish embryo.** Representative, wt SLC35B3 minigene microinjected,1 day-old embryo exhibiting extensive expression of EGFP (ventral view). Y: yolk sack; T: tail; H: head.
