# Supplementary material for: The “Alternative” Choice of Constitutive Exons throughout Evolution
Source: PLoS Genet. 2007 Nov 16;3(11):e203. doi: 10.1371/journal.pgen.0030203 (PMC2077895; doi:10.1371/journal.pgen.0030203)
Supplement: Supplementary file 10 [file pgen.0030203.sg004.doc]

**Fig. S4 Splicing assays on putative BS mutants**. (**A**) The sequence of the 3' end of the eleven intron of IMP gene is shown. The positions of the 3'ss-AG, PPT and the putative BS are shown in bold, italic and red-bold, respectively. Seven positions marked 1 to 7 were mutated from A to G and are shown in red. (B) The wild-type or mutant was introduced into 293T cells by transfection; total RNA was extracted and splicing products were separated in 2% agarose gel, after reverse transcription polymerase chain reaction (RT–PCR). Lane 1 represents splicing products of wild-type (wt) mini-gene and lanes 2-8 represents splicing products of mutated mini-genes. The mini-gene mRNA isoforms are shown on the right: the black box defines the middle exon and the empty boxes define the flanking exons. All cDNA products were confirmed by DNA sequencing.
