# Supplementary material for: The “Alternative” Choice of Constitutive Exons throughout Evolution
Source: PLoS Genet. 2007 Nov 16;3(11):e203. doi: 10.1371/journal.pgen.0030203 (PMC2077895; doi:10.1371/journal.pgen.0030203)
Supplement: Supplementary file 11 [file pgen.0030203.st001.doc]

**Table S1. p-values attributed to the different characteristic between constitutive, alternative and species specific exons.**

| **Parameter** | **Constitutive (C)** | **Species Specific (SS)** | **Alternative (A)** | **P Value (A-C)** | **P Value (C-SS)** | **P Value**  **(A-SS)** |
| --- | --- | --- | --- | --- | --- | --- |
| % identity^1^ | 87.8% | 88.3% | 92.2% | 6×10^-85^ | 0.04 | 6×10^-34^ |
| Length^1^ | 132.5 bp | 126.9 bp | 97.7 bp | 1.1×10^-50^ | 1.4×10^-05^ | 1.78×10^-15^ |
| % symmetrical^2^ | 39.8% | 39.7% | 66.4% | 2.2×10^-39^ | NS | 2.6×10^-20^ |
| ΔG^1^ | -6.06 | -5.93 | -5.62 | 8.8×10^-07^ | NS | 0.013 |
| Ka/Ks^2^ | 0.087 | 0.125 | 0.190 | ≈0 | 1.6×10^-114^ | ≈0 |
| Ks^2^ | 0.495 | 0.428 | 0.237 | ≈0 | 3.2×10^-100^ | ≈0 |
| Fail Ka/Ks^2^ | 7.6% | 13.5% | 33.3% | 8.4×10^-50^ | 3.6×10^-6^ | 4.4×10^-13^ |
| ESR Cons.^2^ | 52.3% | 52.4% | 65.9% | 9.6×10^-92^ | NS | 3.2×10^-56^ |
| Stretch up^1^ | 39.9 bp | 50.9 bp | 76.0 bp | 5.7×10^-40^ | 4×10^-8^ | 4.4×10^-10^ |
| Stretch down^1^ | 37.2 bp | 46.6 bp | 68.2 bp | 1.1×10^-25^ | 0.0025 | 5.5×10^-8^ |
| Cons. up^1^ | 81.1% | 81.5% | 85.2% | 1.7×10^-27^ | NS | 2×10^-12^ |
| Cons. down^1^ | 81.1% | 81.5% | 85.2% | 8.5×10^-11^ | NS | 1.2×10^-4^ |
| Intron up^1^ | 5204.2 bp | 4759.8 bp | 5867.8 bp | 4.1×10^-07^ | NS | 0.012 |
| Intron down^1^ | 4239.2 bp | 3589.2 bp | 4990.6 bp | 2.7×10^-11^ | NS | 2×10^-05^ |
| Inclusion^1^ | 100% | 79.4% | 64.4% | ≈0 | 2.8×10^-8^ | ≈0 |

^1^**P values were calculated with the Mann-Whitney test (2 tails)**

^2^ **P value was calculated using** χ^2^
