# Supplementary material for: The “Alternative” Choice of Constitutive Exons throughout Evolution
Source: PLoS Genet. 2007 Nov 16;3(11):e203. doi: 10.1371/journal.pgen.0030203 (PMC2077895; doi:10.1371/journal.pgen.0030203)
Supplement: Supplementary file 12 [file pgen.0030203.st002.doc]

**Table S2. Accession number for the genes used through this manuscript.**

| **NAME** | **NM** | **Full Name** |
| --- | --- | --- |
| DDO | a - NM_004032  b - NM_003649 | D-aspartate oxidase isoform a/b |
| POLB | NM_002690 | polymerase (DNA directed), beta |
| RABL5 | NM_022777 | RAB, member RAS oncogene family-like 5 |
| MTHFD2 | NM_006636 | methylene tetrahydrofolate dehydrogenase 2 |
| DGCR6L | NM_033257 | DiGeorge syndrome critical region gene 6 like |
| DPM1 | NM_003859 | dolichyl-phosphate mannosyltransferase |
| SLC25A26 | NM_001009938 | solute carrier family 25, member 26 isoform c |
| APGL5 | NM_004849 | autophagy 5-like- |
| SLC35B3 | NM_015948 | solute carrier family 35, member B3 |
| TOM1 | NM_005488 | target of myb1 |
