# Supplementary material for: The “Alternative” Choice of Constitutive Exons throughout Evolution
Source: PLoS Genet. 2007 Nov 16;3(11):e203. doi: 10.1371/journal.pgen.0030203 (PMC2077895; doi:10.1371/journal.pgen.0030203)
Supplement: Supplementary file 13 [file pgen.0030203.st003.doc]

**Table S3. The differences between low inclusion and high inclusion alternative exons' groups as descibes graphically in Fig. 3.**

| **Parameter** | **Low Inclusion (LI)** | **High Inclusion (HI)** | **P Value** | **Test** |
| --- | --- | --- | --- | --- |
| % Identity | 94.22 | 89.75 | 6.8×10^-09^ | Mann-Whitney-2 tails |
| Symmetrical | 87.42 | 50.34 | 3.8×10^-12^ | Chi-Test |
| 5'ss score | 79.69 | 81.83 | 0.016 | Mann-Whitney-2 tails |
| Length | 82.77 | 98.51 | 3×10^-04^ | Mann-Whitney-2 tails |
| % Failure of Ka/Ks test | 45.59 | 22.83 | 1.0×10^-03^ | Chi Test |
| Ka/Ks value | 0.33 | 0.14 | 4.3×10^-66^ | Chi Test |
| Intron conservation (up) | 61.28 | 52 | 0.028 | Mann-Whitney-2 tails |
| Size of stretch (up) | 56.34 | 37.87 | 0.0057 | Mann-Whitney-2 tails |
| Intron conservation (down) | 64.10 | 61.22 | 0.0129 | Mann-Whitney-2 tails |
| Size of stretch (down) | 62.64 | 49.60 | NS | Mann-Whitney-2 tails |
