# Supplementary material for: The “Alternative” Choice of Constitutive Exons throughout Evolution
Source: PLoS Genet. 2007 Nov 16;3(11):e203. doi: 10.1371/journal.pgen.0030203 (PMC2077895; doi:10.1371/journal.pgen.0030203)
Supplement: Supplementary file 14 [file pgen.0030203.st004.doc]

**Table S4. The five putative SR binding sites and their different score among species.**

These five were selected based on three criteria: (1) no overlap with other ESRs (2) a score of at least 0.5, and (3) a difference of at least 1 in ESR score between human and a non mammalian vertebrate. The ESR score is the predicted binding strength to the SR protein as computed using ESEfinder (Cartegni et al. 2003). These criteria increase the chance that the five analyzed ESRs are associated with the shift from constitutive exon in non-mammalian vertebrates to alternative exon in mammals.

**(A)**

| # | SR Family | Human/Mouse | | Chicken | | Zebrafish | |
| --- | --- | --- | --- | --- | --- | --- | --- |
|  |  | sequence | score | sequence | score | sequence | score |
| A | SC35 | GTACTATG | 2.76 | GAACTATG | 3.04 | GGACCATG | 4.16 |
| B | SRp40 | CTTCAAG | 4.04 | CTTCAAG | 4.04 | CTTTAAG | 1.66* |
| C | SC35 | GATTCCTG | 5.29 | GATTCCAG | 4.27 | CATTCCTG | 3.26 |
| D | SF2 | AGGAGGA | 2.79 | AGGCGGG | 1.07* | CGGAGGA | 5.29 |
| E | SRp40 | ATTCAAG | 3.35 | ATACAAG | 4.39 | ATACAAG | 4.39 |

- * Values under the ESEFinder threshold.
- Changes in the binding sites are marked with gray and are with respect to the human sequences.

**(B) The score summery of all ESR according to the mutations that were done following figure 4. for every mutation or combinatorial mutations the overall score according to ESEfinder was calculated**

| Human/Mouse score for A+B+C+D+E = 18.23 | | |
| --- | --- | --- |
| Combination | Human/Mouse into Zebrafish score | Human/Mouse into Chicken score |
| A | 19.63 | 18.51 |
| B | 15.85 | 18.23 |
| C | 16.2 | 17.21 |
| D | 20.73 | 16.51 |
| E | 19.27 | 19.27 |
| A+D | 22.16 | 16.79 |
| B+D | 18.35 | 16.51 |
| A+D+E | 23.17 | 17.83 |
| A+C+D | 20.1 | 15.77 |
| A+B+C+D+E | 18.76 | 16.81 |
