# Supplementary material for: The “Alternative” Choice of Constitutive Exons throughout Evolution
Source: PLoS Genet. 2007 Nov 16;3(11):e203. doi: 10.1371/journal.pgen.0030203 (PMC2077895; doi:10.1371/journal.pgen.0030203)
Supplement: Supplementary file 15 [file pgen.0030203.st005.doc]

**Table S5. List of potential SR binding sites that were found in exon 5 of the human SLC35B gene.**

| **Sequence** | **Type and score** | | **Start** | **End** | **Reason of Exclusion / chosen** |
| --- | --- | --- | --- | --- | --- |
| GTACTATG | SC35 | | 45 | 52 | chosen |
| CTTCAAG | SRp40 | | 103 | 109 | chosen |
| GATTCCTG | SC35 | | 121 | 128 | chosen |
| AGGAGGA | SF2/ASF | | 136 | 142 | chosen |
| ATTCAAG | SRp40 | | 149 | 155 | chosen |
| CTACATG | SRp40 | | 16 | 22 | The difference between the human, chicken and Zebrafish is less than 1. In addition this sequence overlaps with another binding site. |
|  | Human | 3.64 |  |  |  |
|  | Chicken | 3.64 |  |  |  |
|  | zebrafish | 4 |  |  |  |
| TACATG | SRp55 | | 17 | 22 | No difference between human, chicken and zebrafish. In addition this sequence overlaps with another binding site. |
|  | Human | 3.15 |  |  |  |
|  | Chicken | 3.15 |  |  |  |
|  | zebrafish | 3.15 |  |  |  |
| TGGGTA | SRp55 | | 42 | 47 | Exists only in human and overlaps with another binding site. |
|  | Human | 2.71 |  |  |  |
|  | Chicken | - |  |  |  |
|  | zebrafish | - |  |  |  |
| GGGTACTA | SC35 | | 43 | 50 |  |
|  | Human | 2.71 |  |  |  |
|  | Chicken | - |  |  |  |
|  | zebrafish | - |  |  |  |
| GGCTACCT | SC35 | | 74 | 81 | Not Exists in chicken and the change in the score between human and zebrafish is less than 1. |
|  | Human | 2.84 |  |  |  |
|  | Chicken | - |  |  |  |
|  | zebrafish | 2.47 |  |  |  |
| CCCAAGT | SF2/ASF | | 93 | 99 | The difference between human, chicken and Zebrafish is less than 1. |
|  | Human | 2 |  |  |  |
|  | Chicken | 2.6 |  |  |  |
|  | zebrafish | 2 |  |  |  |
| TGCTGC | SRp55 | | 110 | 115 | Chicken has score under 0.5 and no change is found in the score between human and zebrafish. |
|  | Human | 3.02 |  |  |  |
|  | Chicken | 0.46 |  |  |  |
|  | zebrafish | 3.02 |  |  |  |
