# Supplementary material for: The “Alternative” Choice of Constitutive Exons throughout Evolution
Source: PLoS Genet. 2007 Nov 16;3(11):e203. doi: 10.1371/journal.pgen.0030203 (PMC2077895; doi:10.1371/journal.pgen.0030203)
Supplement: Supplementary file 16 [file pgen.0030203.st006.doc]

**Table S6: Ka/Ks analysis of species specific**

Ka/Ks results for the two groups of the species specific

|  | number | A | S | Ka/Ks | Ka | Ks | %identity |
| --- | --- | --- | --- | --- | --- | --- | --- |
| 5'ss changed | 320 | 12267.7 | 4715.3 | 0.1621 | 0.0677 | 0.4178 | 87.1% |
| 5'ss unchanged | 276 | 10621.4 | 4393.6 | 0.1761 | 0.0526 | 0.2984 | 89.6% |

Ka/Ks values after splitting the exons into two

| SS | position | A | S | Ka/Ks | Ka | Ks | p-value | Identity | p-value |
| --- | --- | --- | --- | --- | --- | --- | --- | --- | --- |
| 5'ss changed | close to the 5'ss | 7067.9 | 2874.1 | 0.2533 | 0.0879 | 0.347 | 0.000816 | 86.7% | 0.156 |
|  | Far from the 5'ss | 7050.4 | 2849.6 | 0.292 | 0.0866 | 0.296 |  | 87.5% |  |
| 5'ss  unchanged | close to the 5'ss | 6437.1 | 2667.9 | 0.3088 | 0.0726 | 0.2351 | 0.1637 | 89.8% | 0.325 |
|  | Far from the 5'ss | 5848.6 | 2470.4 | 0.2598 | 0.0665 | 0.256 |  | 89.4% |  |
