# Supplementary material for: The “Alternative” Choice of Constitutive Exons throughout Evolution
Source: PLoS Genet. 2007 Nov 16;3(11):e203. doi: 10.1371/journal.pgen.0030203 (PMC2077895; doi:10.1371/journal.pgen.0030203)
Supplement: Supplementary file 17 [file pgen.0030203.st007.doc]

**Table S7. – two different groups of alternative 5'ss and their ESE/ESS ratio**

| **Type** | **Splicing Pattern** | **ESEs** | **ESSs** | **ESE/ESS ratio** |
| --- | --- | --- | --- | --- |
| Alternative 5’ss have lower 5’ss score | Alternative | 994 | 404 | 2.46 |
|  | Constitutive | 1074 | 337 | 3.19 |
| Alternative 5’ss have same score as constitutive ortholog | Alternative | 1563 | 536 | 2.92 |
|  | Constitutive | 1534 | 483 | 3.18 |
